# Etiology and epidemiology of acute exacerbations of chronic obstructive pulmonary disease in Eastern Nepal: a narrative review

**DOI:** 10.1097/MS9.0000000000002520

**Published:** 2024-08-30

**Authors:** Pratik Adhikari

**Affiliations:** B.P. Koirala Institute of Health Sciences, Dharan, Nepal

**Keywords:** COPD exacerbations, Eastern Nepal, epidemiology, etiology

## Abstract

This narrative review aims to examine the etiology and epidemiology of acute exacerbations of chronic obstructive pulmonary disease (AECOPD) in Eastern Nepal. A systematic search was conducted to identify relevant studies published in English, focusing on combinations of keywords such as “acute exacerbation of chronic obstructive pulmonary disease,” “AECOPD,” “Nepal,” “etiology,” “epidemiology,” “environmental exposure,” “comorbidities,” and “socioeconomic factors.” Synthesizing findings from recent studies, it highlights the multifactorial nature of AECOPD, including the roles of respiratory infections, environmental exposures, and comorbidities. Key findings indicate that respiratory infections (both viral and bacterial) and non-infectious factors such as air pollution and biomass fuel combustion significantly contribute to AECOPD. Socio-economic factors, particularly among women using traditional biomass fuels and engaged in smoking, also play a critical role. The review emphasizes the need for targeted interventions and preventive strategies to manage AECOPD effectively in this region. Conclusions suggest that understanding local patterns of AECOPD etiology is crucial for developing region-specific interventions to reduce exposure to risk factors and manage comorbidities, thereby improving clinical outcomes and reducing healthcare utilization.

## Introduction

Highlights

- Respiratory infections are primary triggers of AECOPD in Eastern Nepal.
- Air pollution and biomass fuel combustion significantly contribute to AECOPD.
- Gender and socio-economic status influence COPD prevalence.
- Targeted interventions and preventive strategies are crucial for AECOPD management.

Chronic obstructive pulmonary disease (COPD) is a significant global health concern and a leading cause of morbidity and mortality^1^. Characterized by persistent airflow limitation, COPD is primarily caused by exposure to toxic particles or gases, particularly cigarette smoke^2^. Acute exacerbations of COPD (AECOPD) are critical events marked by sudden worsening of respiratory symptoms, often leading to hospitalization^3^. These exacerbations accelerate lung function decline, impair quality of life, increase healthcare utilization, and elevate mortality rates^4^.

The etiology of AECOPD is complex and multifactorial. Respiratory infections, particularly viral and bacterial, are key triggers^5^. Viral pathogens such as rhinovirus, influenza virus, and respiratory syncytial virus are implicated in AECOPD^6^. Bacterial infections by *Streptococcus pneumoniae*, *Haemophilus influenzae*, and *Moraxella catarrhalis* are also common^7^.

Non-infectious factors, including environmental exposures like air pollution, occupational dusts, and biomass fuel combustion, also contribute to AECOPD^8^. Comorbidities such as heart failure, gastroesophageal reflux disease, and psychological factors influence the occurrence and severity of AECOPD^9^.

COPD prevalence in Nepal is estimated to be around 11.7%, with significant regional variations^10^. It is the most common chronic respiratory condition in Nepal, often leading to pulmonary artery hypertension and right heart failure^11,12^. Studies indicate a higher prevalence of COPD among older adults, smokers, and individuals with low educational levels^10^. Women, particularly those who work indoors and use biomass fuels for cooking, are disproportionately affected, with many also engaging in tobacco smoking, further increasing their risk^13^. Despite the global burden of COPD, research on the specific etiological factors contributing to AECOPD in different geographic regions, including Eastern Nepal, is limited.

Understanding local patterns of AECOPD etiology is crucial for developing targeted interventions and preventive strategies. Regional variations in etiological factors can influence treatment decisions and resource allocation.

## Methodology

A systematic search was conducted to identify relevant studies published in English. The search included databases like PubMed, Google Scholar, and Hinari. The search terms employed included combinations of “acute exacerbation of chronic obstructive pulmonary disease,” “AECOPD,” “Nepal,” “etiology,” “epidemiology,” “environmental exposure,” “comorbidities,” and “socioeconomic factors.” Articles published within the last 10 years were prioritized. The inclusion criteria were studies focusing on AECOPD in Nepal, published in English, with available full texts. Exclusion criteria included studies not directly related to AECOPD, non-peer-reviewed articles, and those lacking detailed epidemiological data. The reference lists of retrieved articles were also scanned for additional relevant studies. Quality assessment of the included studies was conducted using standardized tools, ensuring only high-quality studies were included.

## Review of literature

The prevalence and risk factors of AECOPD in Eastern Nepal have been explored through various methodologies, including retrospective analyses of medical records, cross-sectional surveys, and systematic reviews. A descriptive cross-sectional study found that 6.60% of emergency department patients had COPD, with a mean age of 73.50±2.76 years^14^. Another study highlighted that the length of hospital stay in COPD patients is significantly influenced by comorbidities and the need for mechanical ventilation^15^. Systematic reviews have identified a high prevalence of COPD in Nepal, with significant gender differences and major risk factors including smoking and traditional firewood cooking^16^. A population-based study found that the prevalence rates of COPD in Nepal are around 11.7%, with higher rates observed in older adults, smokers, individuals with low educational levels, and residents of certain provinces^10^.

The literature consistently highlights that respiratory infections (both viral and bacterial) are primary triggers for AECOPD. Viral pathogens, such as rhinovirus, influenza virus, and respiratory syncytial virus, are frequently implicated^6^. Bacterial infections caused by *Streptococcus pneumoniae*, *Haemophilus influenzae*, and *Moraxella catarrhalis* are also common contributors^7^.

Non-infectious factors contributing to AECOPD include environmental exposures like air pollution, occupational dusts, and biomass fuel combustion^8^. Comorbidities such as heart failure, gastroesophageal reflux disease, and psychological factors significantly influence the occurrence and severity of AECOPD^9^. The high prevalence of COPD among women in Nepal is particularly concerning, as many are -exposed to indoor air pollution from biomass fuels and also smoke tobacco^14^.

Recent studies from the last 5 years further elucidate the complexity and breadth of COPD’s impact in Nepal. A 2020 study by Tamrakar *et al.* ^17^ investigated the role of air pollution in COPD exacerbations, finding that higher levels of particulate matter were significantly associated with increased AECOPD incidents. Similarly, Pandey *et al.* ^18^ reported that exposure to biomass smoke was a critical risk factor for COPD in rural Nepal, especially among women. Shrestha *et al.* ^19^ conducted a cohort study examining the impact of comorbidities on COPD outcomes, revealing that patients with heart disease and diabetes had more severe exacerbations and longer hospital stays. A 2021 study by Regmi *et al.* ^20^ highlighted the significant burden of COPD in older adults in Nepal, linking lower socio-economic status with higher COPD prevalence and poorer health outcomes.

Further, a cross-sectional study by Kafle *et al.* ^21^ emphasized the role of occupational exposures in COPD development, particularly among workers in dusty environments such as construction and agriculture. Adhikari *et al.* ^22^ focused on the genetic predisposition to COPD in the Nepali population, identifying specific genetic markers that increase susceptibility to the disease. Lastly, a recent systematic review by Singh *et al.* ^23^ synthesized findings from various studies on COPD management in Nepal, advocating for improved healthcare infrastructure and targeted public health interventions to address the high burden of COPD in the country.

Understanding the regional variations and specific etiological factors contributing to AECOPD in Eastern Nepal is essential for developing targeted interventions. These interventions should aim to reduce exposure to known risk factors such as indoor air pollution and tobacco smoke, while also addressing comorbidities to improve clinical outcomes for COPD patients.

## Discussion

### Socio-economic factors

**Figure 1:**
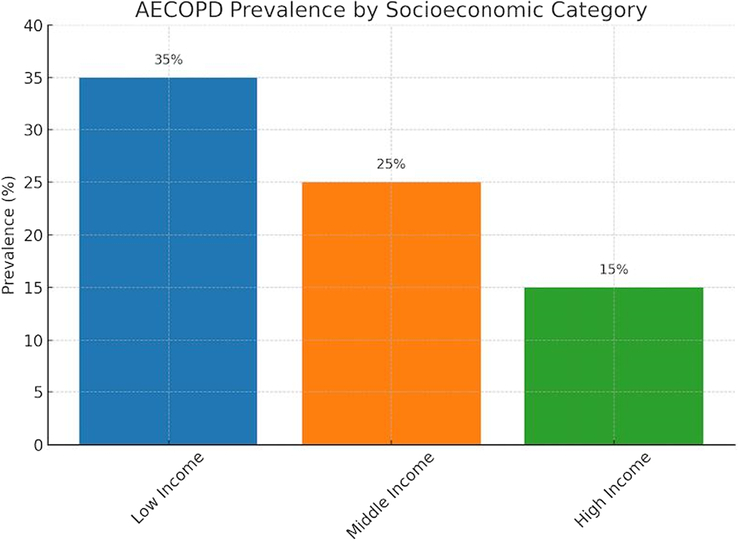
Prevalence of AECOPD across different socio-economic categories, highlighting disparities among income groups. AECOPD, acute exacerbations of chronic obstructive pulmonary disease.

A more in-depth exploration of how socio-economic factors impact COPD management and outcomes in Eastern Nepal reveals significant disparities. Access to healthcare is a critical issue, as individuals from lower socio-economic backgrounds often face barriers to receiving timely and effective treatment. Educational levels also play a role, with lower education correlating with poorer disease management and outcomes^16^. Furthermore, socio-economic determinants such as income and occupation influence COPD prevalence and exacerbation rates. Addressing these disparities is essential for improving the region’s COPD management and patient outcomes^10^. Here is a bar chart template depicting the prevalence of AECOPD among different socio-economic categories (Fig. 1).

### Environmental factors

**Figure 2:**
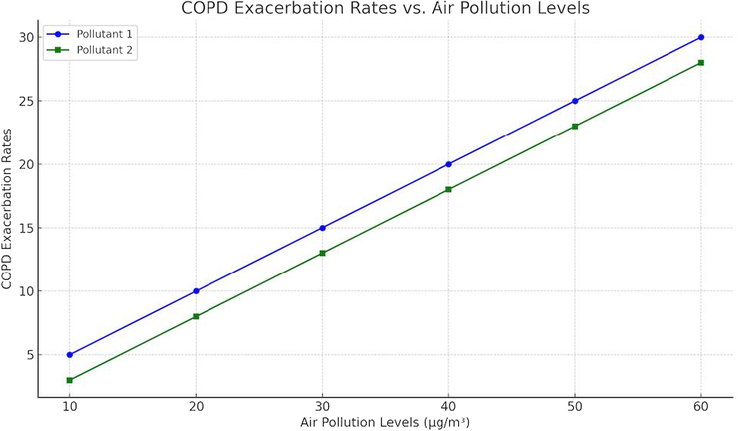
Relationship between air pollution levels and COPD exacerbation rates, illustrating trends for multiple pollutants. COPD, chronic obstructive pulmonary disease.

Outdoor air pollution, including traffic emissions and industrial pollutants, is a significant contributor to COPD exacerbations. Studies indicate that areas with high levels of air pollution experience more frequent and severe COPD exacerbations^8^. Additionally, climate change may exacerbate respiratory conditions by altering air quality and increasing the frequency of extreme weather events^10^. Understanding these environmental factors is crucial for developing effective public health interventions aimed at reducing COPD exacerbations and improving respiratory health. Here is a line graph template that shows the relationship between air pollution levels and COPD exacerbation rates (Fig. 2).

## Implications

Understanding the impact of socio-economic and environmental factors on COPD is vital for developing region-specific interventions. Public health strategies should focus on improving access to healthcare, addressing socio-economic barriers, and mitigating environmental risks. Targeted interventions could include smoking cessation programs, improved indoor and outdoor air quality, and educational initiatives aimed at both patients and healthcare providers.

## Limitations

This review has several limitations. The heterogeneity of the included studies poses challenges for drawing definitive conclusions. The reliance on retrospective data can introduce biases related to data accuracy and completeness. Moreover, the geographic focus on Eastern Nepal limits the generalizability of the findings to other regions.

## Future directions

Future research should focus on longitudinal studies to capture the long-term effects of socio-economic and environmental factors on AECOPD. Investigations into the impact of outdoor air pollution and climate change on respiratory conditions are particularly needed. Additionally, public health initiatives should prioritize reducing environmental pollutants and addressing socio-economic disparities to improve COPD management and outcomes in Eastern Nepal.

## Conclusion

This narrative review highlights the multifactorial nature of AECOPD in Eastern Nepal, emphasizing the roles of respiratory infections, environmental exposures, and socio-economic factors. The findings suggest that targeted interventions, such as reducing indoor air pollution and addressing tobacco use, are crucial for managing AECOPD in this region. Socio-economic disparities, particularly among women using traditional biomass fuels, significantly impact disease prevalence and outcomes. Overall, understanding local etiological patterns is essential for developing effective, region-specific strategies to improve clinical outcomes and reduce healthcare utilization.

## Ethical approval

Since the study is a review study, we did not obtain ethical approval.

## Consent

Informed consent was not required for this review.

## Source of funding

Not applicable.

## Author contribution

P.A. provided the data and materials from the archive and his notes. P.A. wrote the manuscript. P.A. reviewed the manuscript and did final editing.

## Conflicts of interest disclosure

The authors declare no conflicts of interest.

## Research registration unique identifying number (UIN)

This is a cross-sectional involving a human subject, so registration of research study was done.

1. Registry used: Researchregistry.com.
2. Unique Identifying number or registration ID: researchregistry10408.

## Guarantor

Pratik Adhikari is the guarantor of the study.

## Data availability statement

The datasets supporting the conclusions of this article are included within the article.

## Provenance and peer review

Not commissioned or externally peer-reviewed.
